# PEA Electromagnetic Distortion Reduction by Impedance Grounding and Pulsed Voltage Electrode Configurations

**DOI:** 10.3390/s21175837

**Published:** 2021-08-30

**Authors:** Guillermo Mier Escurra, Armando Rodrigo Mor, Luis Carlos Castro, Peter Vaessen

**Affiliations:** 1 High Voltage Laboratory, DCE&S, Department Electrical Sustainable Energy, Delft University of Technology, Mekelweg 4, 2628 CD Delft, The Netherlands or arrodmor@ite.upv.es (A.R.M.); L.C.CastroHeredia@tudelft.nl (L.C.C.); Peter.Vaessen@kema.com (P.V.); 2 Instituto de Tecnología Eléctrica, Universitat Politècnica de València, 46022 Valencia, Spain; 3 KEMA Laboratories, Klingelbeekseweg 195, 6812 DE Arnhem, The Netherlands

**Keywords:** space charges, pulse electro-acoustic method (PEA), electromagnetic compatibility (EMC), high voltage cables, piezoelectric sensor, dielectrics

## Abstract

Space charges are one of the main challenges facing the constantly increasing use of extruded high voltage direct current (HVDC) cables. The Pulsed Electro-Acoustic (PEA) method is one of the most common procedures for space charge measurements of insulation. One issue with the PEA method is distortion due to the crosstalk between the applied voltage pulse and the acoustic sensor. This work analyzed two factors involved in the reduction in this distortion: the influence of the exposed semiconductor distance between the injection electrodes and PEA test cell, and the influence of adding a reactance at the grounding circuit of the PEA test cell. The interaction of these two factors with the distortion was analyzed through a series of experimental testing. Moreover, the performance regarding distortion after applying a developed coaxial injection was compared with the standard non-coaxial injection configuration. It was observed that these two factors had a direct impact on distortion and can be utilized for the reduction in distortion arising from the crosstalk of the applied pulsed voltage. The results can be utilized for the consideration of practical aspects during the construction of a PEA test setup for the measurement of full-size HVDC cables.

## 1. Introduction

With the increasing use of extruded power cables for HVDC systems, the use of methods measuring space charge phenomena has become crucially important. It is well known that space charge phenomena are one of the main challenges facing the development of extruded HVDC cables, as space charges in dielectrics distort electric fields, producing high localized stresses [1,2,3] and influencing the dielectric behavior of dielectric materials [4].

In [5], it was observed that the local enhancement due to space charges led to electrical treeing and, finally, failure. The authors of [6] used a thermoelectrical model to analyze the aging by space charge phenomena in dielectric materials. In [7], the role of space charges in multilayer epoxy commonly utilized in power electronics was studied. The authors of [8] analyzed the aging that occurs during the de-trapping of space charges. Articles [9,10,11] present different aging and life models and their relation with space charge phenomena. The effect of aging in space charge behavior was analyzed in [12] for epoxy and polyethylene, and for XLPE HVDC cable insulation in [13,14]. Promising HVDC cable materials were the focus of [15]. Put briefly, space charge behavior in solid dielectrics impacts upon the reliability of HVDC cable systems.

Nowadays, several non-destructive methods (where dielectric material is not destroyed) of measuring space charge distribution are available; for literature reviews, see [16,17,18,19]. Of these methods, the acoustic and thermal methods are the most common, non-destructive, means of space charge measurement in HVDC solid dielectrics. Though much research has been conducted with the intent of improving measures, most research has studied flat samples and mini cables due to difficulties associated with full-size cable measurement Nevertheless, successful space charge measurements have been performed since the 1990s [20,21,22] and measurement of extruded HVDC cable qualification tests are being performed worldwide [23]. To date, there is an IEEE-proposed protocol for the measurement of space charges for HVDC cables up to 550 kV [24]. The practice of measuring space charges in full-size cables, instead of mini cables, has the advantages of assessing the manufacturing process of cables, and allowing the testing of relationships between a combination of variables that can only be achieved in full-size cables. This includes the combined variables of absolute temperatures and temperature gradients [25], and the relation of insulation thickness with trap distribution characteristics [26].

The aim of this paper is to analyze practical issues in constructing space charge measurement setups utilizing the Pulsed Electro-Acoustic (PEA) method used in full-size HVDC cables.

One of the issues that impacts space charge measurement using the PEA method is the crosstalk between the piezoelectric sensor and the applied pulsed voltage required for the PEA method. The signal distortion affecting measurement due to this crosstalk has the potential to interfere with the useful acoustic signal, which may result in inaccuracies and errors during post-processing.

A common method used to eliminate these spurious signals involves subtracting the reference signal with no space charges or applied HVDC from the subsequent measurements of interest. Nevertheless, this procedure can prove ineffective when the sample to be measured has pre-existing charges, as subtracting disturbance in cases where distortion overlaps with the acoustic signal will also subtract the pre-existing space charge components. In cases of extreme distortion in the signal, the distortion magnitude and duration may also result in an effective reduction in the vertical resolution at the acoustic signal measured at the oscilloscope, even reaching saturation of the electronics.

The authors of [27] analyzed crosstalk interference between the pulsed voltage and acoustic sensor due to the physical location of the pulse injection connection at the electrodes, and the location of the grounding connection. This paper aims to address the same crosstalk issue by analyzing two different factors which influence the generated crosstalk distortion, specifically when the PEA test cell is grounded. The grounding of the PEA test cell might be required due to specific requirements, such as having a continuous measurement during long periods of cable testing, perhaps requiring power to PEA test cell devices over several days.

Two factors were analyzed in this research: first, the influence of the distance of the exposed semiconductor between injection electrodes and the PEA test cell. Second, the influence of adding a reactance at the grounding circuit of the PEA test cell.

Both factors were also tested using an alternative, recently developed, coaxial configuration for the injection of the pulsed voltage. This configuration is described in Section 2.3. The aim of the configuration was to test whether crosstalk was reduced by means of a coaxial injection.

The reduction in crosstalk distortion improved the measured signal. This, in turn, has the potential to reduce errors during post-processing and allow for better interpretation of the measured space charge distribution in the HVDC cables.

This paper is organized as follows: Section 2 describes the utilized test equipment, the HVDC sample cable characteristics, and the different, pulsed voltage, injection configurations that were compared. In Section 3, the results from the test measurements of each configuration are presented, discussed, and compared. Section 4 provides conclusions.

## 2. Experimental Setup

### 2.1. PEA Test Cell

**Figure 1:**
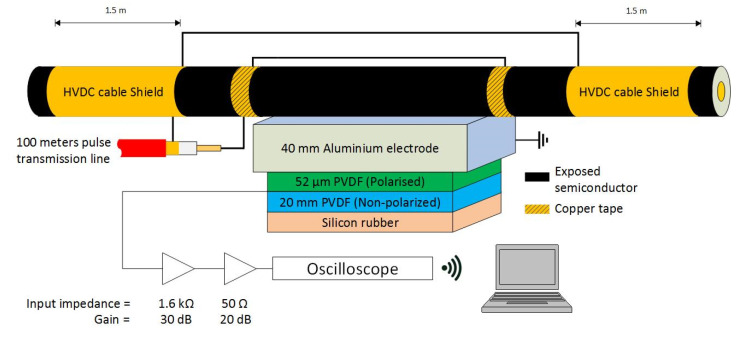
Representation of the PEA test cell including the acoustic sensor, amplifier, and oscilloscope.

The PEA test cell used for the experiments was built while considering the geometry of the cable sample. A diagram illustrating the PEA test cell and the HVDC cable can be seen in Figure 1. The PEA cell used a flat electrode configuration to facilitate an optimum acoustic contact with the cylindrical geometry of the HVDC cable. The electrode consisted of a 40 mm thick aluminum block between the external semiconductor layer of the HVDC cable and the piezoelectric film. The lateral dimensions of the block were 300 mm × 300 mm. These dimensions were selected in view of the acoustic propagation speeds of 6420 m/s and 2000 m/s, for the aluminum and XLPE, respectively. With a cable insulation thickness of 21.5 mm, the minimum thickness of the aluminum electrode required to avoid the overlapping of the signal due to reflections was 35 mm [28].

The acoustic sensor consisted of a 52 µm thick polyvinylidene fluoride (PVDF) piezo film [29], backed with 20 mm of non-polarized PVDF to avoid reflections, and terminated with rubber for damping purposes. The contact area of the piezo film was 5 × 65 mm^2^, with the longest side parallel to the length of the HVDC cable. The capacitance of the piezo was 0.83 nF. The PVDF piezo film was connected to a charge amplifier with 1.6 kΩ input resistance and 30 dB gain in series with a 20 dB amplifier (all were battery powered). The piezo film and the amplifiers were contained in an aluminum box of 400 mm × 200 mm × 120 mm (external dimensions), with 4 mm wall thickness.

The acoustic signal from the amplifiers was measured using a battery-powered oscilloscope with a sampling rate of 125 MS/s and 40 MHz bandwidth. The signals were then averaged 100 times.

The HVDC test cable shield electrodes at each side of the PEA test cell were 1.5 m long, with an approximate capacitance towards the HVDC cable’s conductor of 390 pF each.

### 2.2. Pulsed Voltage Injection

**Figure 2:**
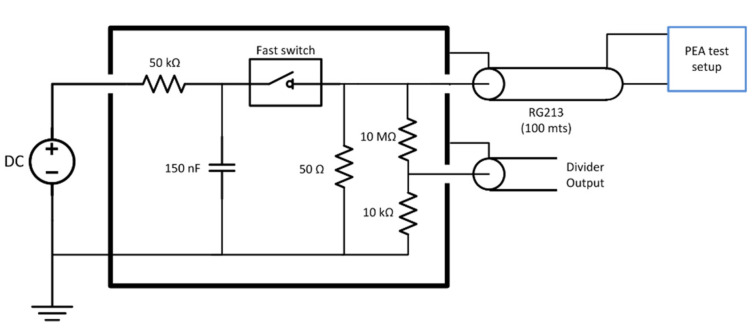
Voltage pulse generator equivalent circuit.

The voltage impulse was generated using a Behlke HTS 61-40 fast switch metal–oxide–semiconductor field-effect transistor (MOSFET) using a total of 150 nF discharge capacitors and a DC source to recharge the capacitors between each pulse. The maximum charging voltage applied was 4 kV DC. The pulse traveled from the switch to the PEA test cell through a coaxial cable (RG213) of 50 Ω characteristic impedance. The coax cable was 100 m long to electrically decouple the switch from the test cell due to the propagation time and the pulse duration. The pulse had a 300 ns duration. The cable was non-terminated at the PEA test setup side to maximize the voltage due to impedance mismatch. The cable was terminated at the pulse generator side to avoid pulse reflections [30]. The equivalent circuit of the pulse generator can be seen in Figure 2.

Experimental sets were performed in two different pulsed voltage injection configurations to observe the difference between a coaxial and noncoaxial structure in relation to the crosstalk occurring at the piezo amplifier at the instant of pulsed voltage injection.

**Figure 3:**
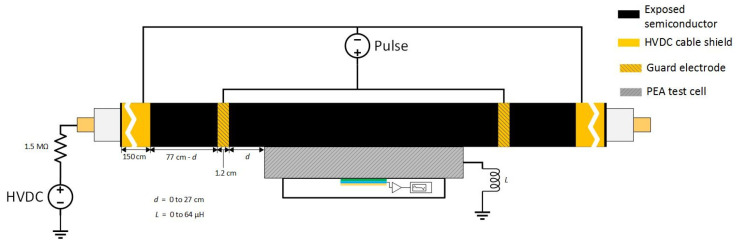
Application of the pulsed voltage at the HVDC test cable using the non-coaxial configuration. “d” stands for the distance between the injection electrode and the PEA test cell. “L” is the applied inductance at the PEA test cell grounding.

The non-coaxial configuration involved a connection between the HVDC cable shield and the guard electrodes. The guard electrodes were situated at each side of the PEA test cell and its distance towards the test cell ranged from 0 cm to 27 cm, depending on the test. The connection was established through a single conductor, as can be seen in Figure 3. In this figure, we can observe the variable “d”, which stands for the distance between the injection electrode and the PEA test cell, as well as the inductance “L” due to the application of N30 ferrites at the PEA test cell grounding.

**Figure 4:**
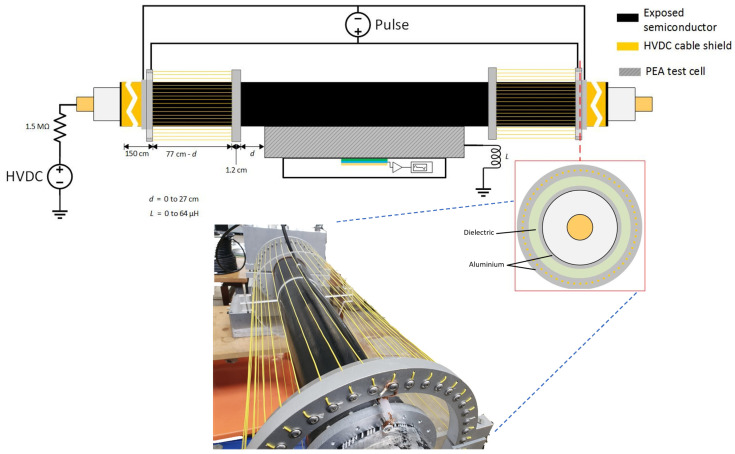
Application of the pulsed voltage at the HVDC test cable using the coaxial configuration. “d” stands for the distance between the injection electrode and the PEA test cell, “L” is the applied inductance at the PEA test cell grounding.

The coaxial injection consisted of an array of 40-line conductors arranged in parallel around the HVDC cable test sample. In this way, the parallel array of cables around the HVDC cable were utilized as the return conductor, while the HVDC cable (semiconductor and internal conductor) acted as the internal conductor of the coaxial structure of the pulsed voltage injection. It is important to mention that the coaxial injection was directly connected to the coaxial transmission cable of 50 Ω, stemming from the pulse generator and joining to the PEA test cell. This ensured that a coaxial structure was retained throughout the whole pulse circuit. The schematic of the injection can be viewed in Figure 4. Just as with the non-coaxial injection, we can observe the variable “d”, which stands for the distance between the injection electrode and the PEA test cell, and the inductance “L” due to the application of N30 ferrites at the PEA test cell grounding.

### 2.3. HVDC Test Cable

For this test, a 320 kV HVDC cable was used as a test object. The cable used copper as the inner conductor, and aluminum as the outer conductor. The dielectric material was cross-linked polyethylene (XLPE). The geometric characteristics of the HVDC cable sample are shown in Table 1.

The outer layers of the HVDC cable sample were removed in the middle of its length to expose the outer semiconductor, and to mechanically fix the cable to the PEA test cell. The semiconductor was kept continuous, meaning that no section was cut or removed to modify its electric continuity between electrodes. The outer semiconductor was in direct contact with the aluminum electrode of the PEA test cell. To ensure a good acoustic contact, silicone oil was used in the interface, and compressive mechanical force was applied using screws.

## 3. Results and Discussion

Throughout this study, two main test sets (described in Section 2.2) were performed. These two sets of tests were performed for the non-coaxial injection configuration and the coaxial injection configuration to observe the impact of the coaxial structure for the pulse injection.

**Figure 5:**
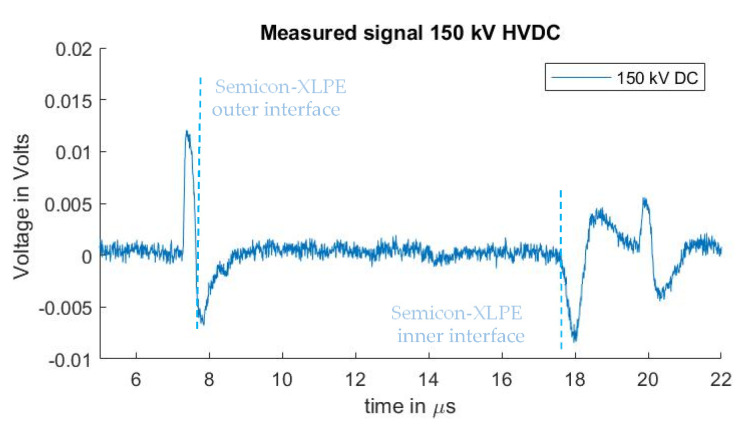
Measured signal with 150 kV HVDC without distortion (by means of subtracting a measure signal with no HVDC applied).

A PEA measurement to be used as a reference for the sensitivity of the measuring system was performed by applying 150 kV at the test sample. This can be observed in Figure 5. The measurement was performed using the non-coaxial injection structure, with 0 cm distance between the guard electrode and the PEA test cell (no ferrites were used). Distortion was eliminated in post-processing by subtracting a measure signal without HVDC applied. While this subtraction procedure can be utilized to observe the interface charges due to the HVDC, the signal of the pre-existing charges is also eliminated and cannot be analyzed in this way, as previously described. For the remaining experiments in Section 3.1 and Section 3.2, no DC voltage was applied to the HVDC cable. Existing space charges can, however, be observed in the reported measurements, as the cable had been previously subjected to HVDC tests that are unrelated to this work.

### 3.1. Non-Coaxial Injection

In these tests, the non-coaxial injection was used. The following test cases were performed to observe the influence of crosstalk between the pulsed voltage and the piezo sensor by varying two parameters:

• The first parameter is the impedance between the electrode guards and the PEA test cell caused by increasing the distance between them and the semiconductor.
• The second parameter is the addition of an impedance for high frequencies at the earthing line of the PEA test cell by means of adding N30 ferrites, as described in Section 2.2.

A summary of the test cases with the non-coaxial injection, the results of which are reported in this work, can be seen in Table 2. In the table, the first column presents the given name to identify the case throughout the paper. In the second column, “L” is the total inductance of the added N30 ferrites. In the third column, “d” stands for semiconductor separation between the PEA test cell and the guard electrodes.

#### 3.1.1. Non-Coaxial Injection at Different Semiconductor Distances

**Figure 6:**
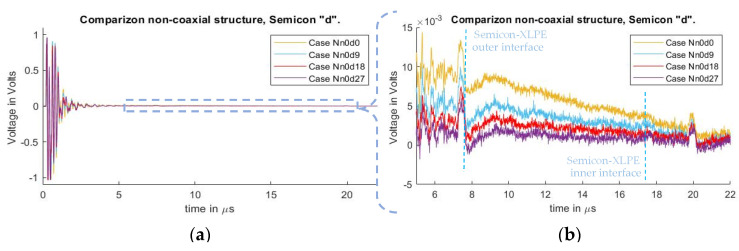
Measured disturbance for cases with different distances “d” for non-coaxial injection. (**a**) Full measured signal ranging from the instant of the pulsed voltage application up to 22 µs. (**b**) Focus on the time instant of the acoustic signal arrival belonging to the charge measurements.

Figure 6 depicts the signals measured in cases Nn0d0, Nn0d9, Nn0d18, and Nn0d27, and which correspond to no added external inductance and differences in semiconductor distances. The whole measured signal, starting at the instant of pulsed voltage application of 0 µs, is illustrated here. At 7.4 µs, the first acoustic peak of the HVDC test cable’s outer electrode can be observed. The acoustic peak of the charges in the inner conductor of the HVDC cable was at 17.9 µs, but due to the small amount of charges, it could not be distinguished. The acoustic peak at 20 µs is due to reflections at the aluminum block of the PEA test cell’s outer electrode acoustic signal.

In Figure 6a, one can observe a distorted signal starting at 0 µs (instant of the applied voltage pulse) due to crosstalk between the pulsed voltage and piezo amplifier. The signal evidences a decaying offset, overlapping with the acoustic signal starting at 7.2 µs. It is also observed that the magnitude of the undesired offset differs between cases as semiconductor distance increases. The reduction in the distorted offset does not follow a linear reduction in relation to the semiconductor distance. Instead, the reduction follows an exponential decay as semiconductor distance between the guarded electrode and the PEA test cell increases.

**Figure 7:**
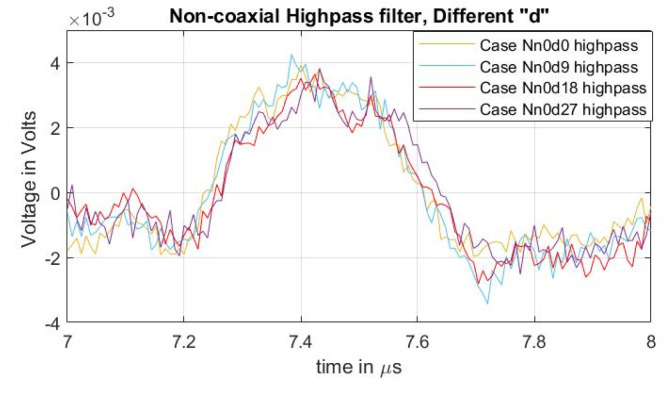
Acoustic magnitude comparison between cases of varying distance “d” for non-coaxial injection.

To compare the acoustic magnitudes between the different semiconductor distances, a high-pass filter with a passband frequency of 500 kHz was applied to the measured signals. The result can be observed in Figure 7, particularly on the first acoustic peak. From the figure, it can be concluded that, while the undesired distorted signal is reduced (Figure 6b), the magnitude of the acoustic signal due to increases in the semiconductor distance “d” did not present a significant impact. In [31], the effects of impedance upon the semiconductor, with respect to the efficiency of the applied pulsed voltage, are presented. In these tests, the increase in “d” reduces the semiconductor distance (and therefore the impedance) between the two injection electrodes (see Figure 3 and Figure 4) meaning that, at higher values of “d”, the acoustic reduction should become more noticeable.

It is important to mention that the procedure of applying the high-pass filter, utilized for the magnitude analysis of Figure 7, is not a recommended practice for eliminating the distortion offset of actual space charge measurements during post-processing. This is because it is possible to lose valuable data about the space charge distribution across the dielectric.

#### 3.1.2. Non-Coaxial Injection with Inductive Ground Path

These test sets were performed to evaluate the impact of grounding impedance utilizing N30 ferrites at the crosstalk distortion between the pulsed voltage and the piezo amplifier (as described in Section 2.2). 

**Figure 8:**
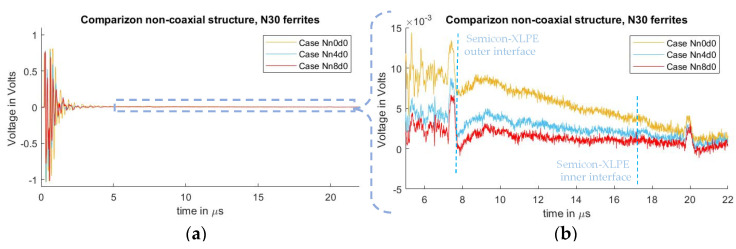
Measured disturbance for cases with different ground inductance “L” for non-coaxial injection. (**a**) Full measured signal ranging from the instant of pulse voltage application up to 22 µs. (**b**) Focus on the time instant of the acoustic signal arrival belonging to the charge measurements.

In Figure 8, the measured signals from cases Nn0d0, Nn4d0, and Nn8d0 can be observed. As seen in the test results from the previous subchapter, one can observe the crosstalk due to the pulsed voltage and the piezo amplifier interaction, where the distorted signal overlaps with the acoustic signal, starting at 7.2 µs. It can also be observed that, by utilizing ferrites at the grounding conductor of the PEA test cell, the distorted signal is diminished, with an exponential decay relation similar to what was observed in the case where semiconductor distance “d” was increased.

**Figure 9:**
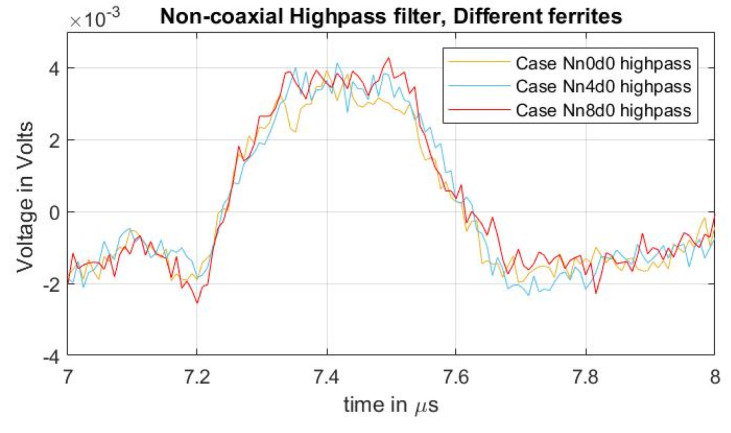
Acoustic magnitude comparison between the different cases of adding external inductance to the ground path.

As in previous cases reported here, a high-pass filter with a passband frequency of 500 kHz was applied to the measured signals to compare the acoustic magnitudes between different quantities of applied ferrites at the grounding conductor. The result can be observed in Figure 9, with a focus on the first acoustic peak. From the figure, it should be noted that, while the undesired distorted signal is reduced (Figure 8b), the magnitude of the acoustic signal did not present a noticeable difference as a result of adding external inductance to the ground path.

#### 3.1.3. Non-Coaxial Injection Utilizing Semiconductor Distance and Inductive Ground Path

**Figure 10:**
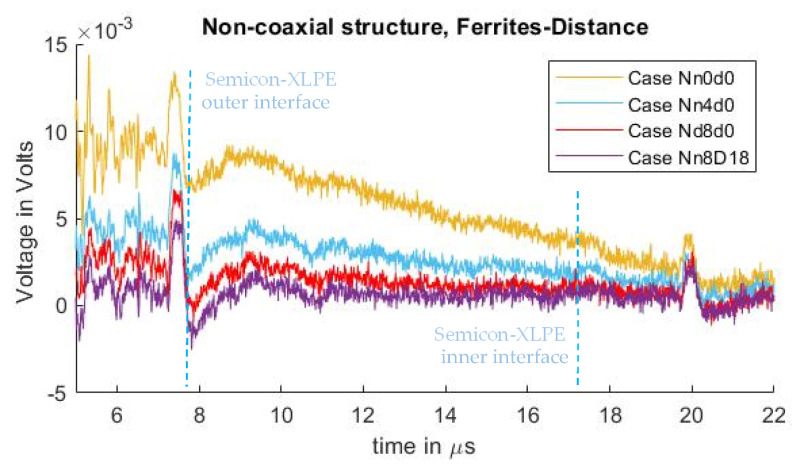
Comparison of measured signals with different semiconductor distances “d” combined with several ground path inductances.

Too large an increase to either the distance between the injection electrode and the PEA test cell, or the level of PEA test cell grounding impedance may not always be feasible. Nevertheless, the combination of both reduction methods can be applied to further reduce distortion. Figure 10 shows the comparison between the effects of applying an incremental number of ferrites at the PEA test cell grounding, and the effects of further reduction by combining ferrites with increased distance between the guard electrode and the PEA test cell.

### 3.2. Coaxial Injection

A second set of experiments utilizing the coaxial structure described in chapter 2.2 were performed to analyze coaxial, pulsed voltage, injection performance on the crosstalk distortion resulting from interaction with the piezo amplifier. These tests were similar to those performed with the non-coaxial injection. The experiments for the coaxial injection can be seen in Table 3. The first column presents the given name to identify the case throughout the paper. In the second column, “L” is the total inductance of the added N30 ferrites and, in the third column, “d” stands for semiconductor separation between the PEA test cell and the guard electrodes.

**Figure 11:**
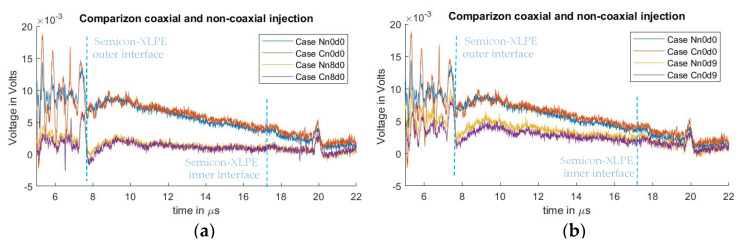
Comparison of acoustic signal distortion utilizing either non-coaxial or coaxial injection. (**a**) Comparison between 0 and 9 cm semiconductor distance “d”. (**b**) Comparison between 0 and 69.6 µH at the PEA test cell grounding “L”.

Figure 11 shows a comparison between the signals measured from the non-coaxial structure condition, and those from the coaxial structure condition. In Figure 11a, the effect of applying eight ferrites to the electrode guard are compared. Figure 11b illustrates the comparison between applying a distance “d” of 9 cm between the PEA test cell and the electrode guard (for the non-coaxial injection) or the aluminum disk (for the coaxial injection). The measurements without applied distance “d”, or ferrites at the electrode guard for the coaxial and non-coaxial structures, are also plotted as reference. One can observe that there is no significant difference in the reduction in crosstalk distortion utilizing coaxial injection as opposed to non-coaxial injection.

## 4. Conclusions

The use of a pulsed voltage in the PEA method produces an electromagnetic transient across the test, yielding a crosstalk signal at the piezo amplifier with a decaying component that overlaps with the relevant acoustic signal used for space charge measurement. This work shows that, by increasing the impedance between the pulsed voltage connection and the ground path at the PEA test cell, the slow decaying component of the crosstalk distortion can be further attenuated.

Increasing the semiconductor distance between the electrode and the PEA test cell or increasing the ground path inductance by adding N30 ferrites at the grounding line successfully decreases the crosstalk that overlaps with the acoustic signal, without significantly impacting acoustic magnitude.

The use of a coaxial injection for the pulsed voltage did not show significant improvement regarding the crosstalk distortion.

The decision of which method to utilize should be informed by the availability of space on the HVDC test cable to expose the semiconductive layer. Regarding inductance impedance at the PEA test cell grounding, one should consider the tradeoff: higher impedance results in less crosstalk but increases the chances of enhanced transient overvoltage in cases of short circuits. At the same time, increasing the semiconductor distance between the PEA test cell and the guard electrodes requires more available HVDC cable length for semiconductor exposure.

The results of the experiments presented in this paper, by means of experimental testing, serve as a guideline for best practices in testing HVDC cable space charge using PEAs that minimize signal distortion and allow for simpler post-processing. The design of a PEA setup should take these factors into consideration given their impact upon crosstalk, especially in configurations where PEA test cells are grounded, and distortion is higher.

## Figures and Tables

**Table 1:** HVDC cable properties.

Property	Value
Inner conductor (diameter)	62.3 mm
Inner semi-conductive layer thickness	1.9 mm
Insulation thickness (XLPE)	21.5 mm
Outer semi-conductive layer thickness	1.5 mm
Exposed semiconductor length	1.5 m
Total cable length	9 m
Cable weight	34.1 kg/m

**Table 2:** Non-coaxial injection test cases.

Case	Added Ground Inductance L	Semiconductor Distance d
Case Nn0d0	0	0 cm
Case Nn0d9	0	9 cm
Case Nn0d18	0	18 cm
Case Nn0d27	0	27 cm
Case Nn4d0	34.8 µH (4 N30 ferrites)	0 cm
Case Nn8d0	69.6 µH (8 N30 ferrites)	0 cm
Case Nn8d18	69.6 µH (8 N30 ferrites)	18 cm

**Table 3:** Coaxial injection test cases.

Case	Applied Ground Inductance L	Semiconductor Distance d
Case Cn0d0	0	0 cm
Case Cn0d9	0	9 cm
Case Cn4d0	34.8 µH (4 N30 ferrites)	0 cm
Case Cn8d0	69.6 µH (8 N30 ferrites)	0 cm
